# Atypical Mongolian Spots With Hurler’s Disease: A Case Report

**DOI:** 10.7759/cureus.58501

**Published:** 2024-04-17

**Authors:** Saranya Athanti, Sadaf Mouzam, Mohammad Sohail Ahmed

**Affiliations:** 1 Pediatrics, Employee State Insurance Corporation Medical College and Hospital, Sanathnagar, Hyderabad, IND

**Keywords:** neurocristopathies, lysosomal storage disorders, mucopolysaccharidosis, hurler’s disease, mongolian spots

## Abstract

Mongolian spots are bluish-grey, irregular, hyperpigmented macules present at birth or that appear in the first few weeks of life. They are classified as atypical if they occur in unusual locations without spontaneous disappearance after infancy; or if new lesions continue to appear beyond early infancy. Although they are generally considered benign, recent studies have shown that atypical Mongolian spots may be associated with inborn errors of metabolism, such as lysosomal storage disorders and neurocristopathies.

An 11-month-old male presented with multiple aberrant Mongolian spots on the abdomen, back, buttocks, arms, and legs, with the largest patch measuring 10x10 cm. Additionally, the child exhibited coarse facial features, a high-arched palate, low-set ears, and a depressed nasal bridge. Systemic examination revealed hepatosplenomegaly, fundus examination showed a hazy cornea, and the urine glycosaminoglycan test was positive, prompting us to conduct further research prioritising lysosomal storage disorders. The mucopolysaccharidosis (MPS) spot test was positive, and electrophoresis for MPS revealed bands for chondroitin sulfate and dermatan sulfate, confirming the diagnosis of MPS. Enzyme assay revealed no alpha-iduronidase activity and normal beta-galactosidase activity, thus confirming Hurler's disease.

This case report highlights the importance of considering atypical Mongolian spots as a potential indicator of underlying storage disorders, enabling early intervention.

## Introduction

Mongolian spots are bluish-grey, irregular, hyperpigmented macules present at birth or that appear in the first few weeks of life. They are most commonly present in the lumbosacral and gluteal regions [1]. They can be single or multiple, round or oval [2]. The blue colour of Mongolian spots is caused by the Tyndall effect, where light scatters due to particles in its path. Dermal pigmentation appears grey or greyish due to shorter wavelengths reflecting from the skin surface, while longer wavelengths like yellow, orange, or red penetrate deeper into the skin. Melanin levels, melanocyte quantity, and their depth in the dermis determine the colour [3].

They occur when melanocytes become trapped in the dermis due to halted transdermal migration, from the neural crest to the epidermis during embryonic development. These lesions naturally begin to fade as dermal melanocytes become encapsulated by extracellular fibrous sheaths [4]. This process begins in utero but is most significant during infancy. They commonly disappear by the age of one year, but some may persist beyond infancy [5].

The term "Mongolian spots" was coined by German professor Edwin Baelz, who first observed them in Mongolian individuals. Mongolian spots are typically more prevalent among newborns of Asian and African descent. They are found approximately in 10% of Caucasians, 50% of Hispanic infants, and 90%-100% of Asian and African infants [2]. Sex and gestational age do not show a significant association [6].

Mongolian spots are classified as atypical if they occur in unusual locations such as the upper back, abdomen, chest, face, arms, and legs; if they are distributed throughout the body; without spontaneous disappearance after infancy; or if new lesions continue to appear beyond early infancy. Although Mongolian spots are generally considered benign, recent studies have shown that atypical Mongolian spots may be associated with inborn errors of metabolism, such as lysosomal storage disorders and neurocristopathies [1].

Weissbluth et al. were the first to acknowledge a link between atypical Mongolian spots and different storage disorders and described it as coincidental. Despite the high prevalence of Mongolian spots in Asians and Africans, the correlation between these two conditions may initially seem coincidental. However, numerous case reports published over the last thirty years have established a connection between them [7].

Hurler's disease is the lysosomal storage disorder most frequently linked with Mongolian spots, with GM1 gangliosidosis being the next most common association. Beyond GM1 gangliosidosis and MPS type I (Hurler’s disease), Mongolian spots have also been observed in cases of MPS type II (Hunter’s syndrome), mucolipidosis, Niemann-Pick disease, and mannosidosis [3].

## Case presentation

An 11-month-old male presented with multiple bluish-grey spots on his buttocks, back, abdomen, arms, and legs since birth. He was born at term via cesarean delivery to parents with third-degree consanguinity, with a birth weight of 2.7 kg. He cried immediately after birth and required no neonatal intensive care unit stay. There was no developmental delay. The child was fully immunised for his age according to the national immunisation schedule.

The past medical history was significant for admission to the pediatric intensive care unit due to a lower respiratory tract infection at the age of 2 months. Additionally, a left inguinal hernia repair was performed 50 days after birth.

**Figure 1:**
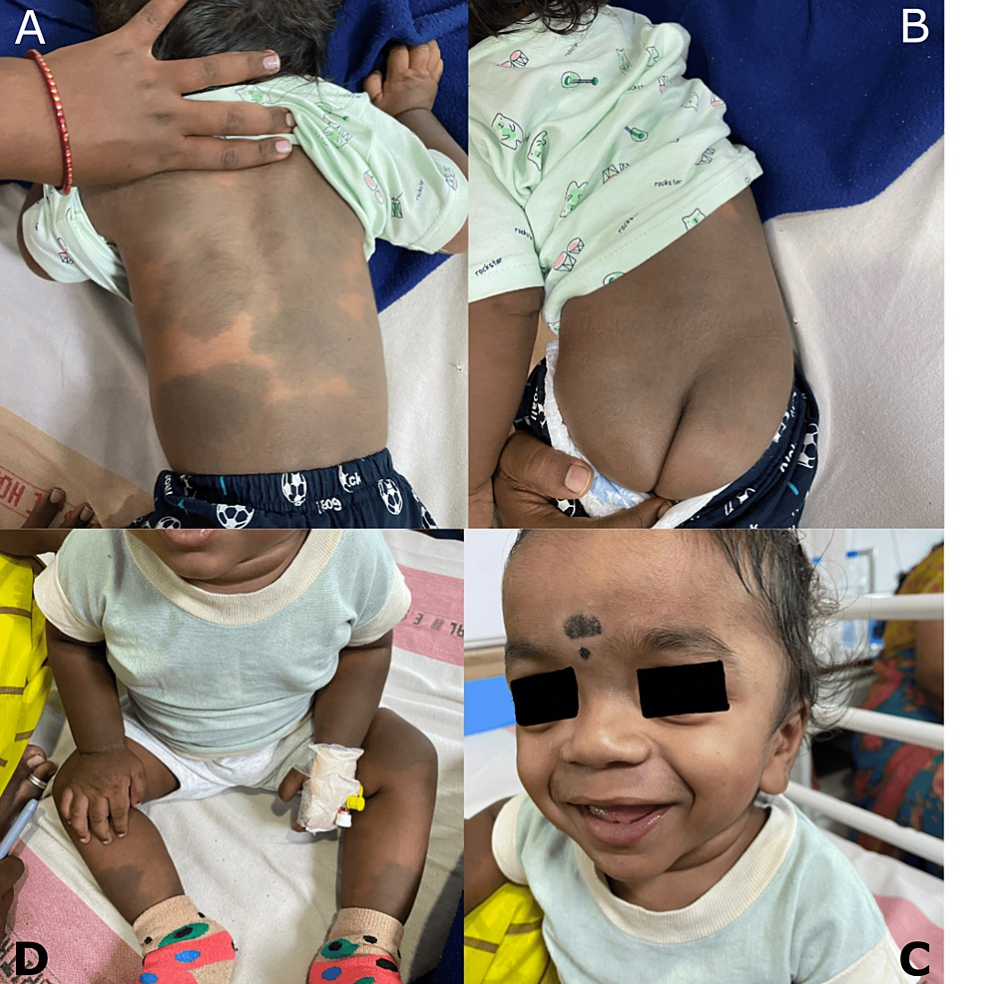
Multiple aberrant Mongolian spots on the back (A), buttocks (B), arms, and legs (D) with coarse facial features, low-set ears, and depressed nasal bridge (C).

During the general examination, the child appeared active, without signs of pallor, icterus, clubbing, cyanosis, generalised edema, or lymphadenopathy. A head-to-toe examination revealed multiple aberrant Mongolian spots on the abdomen, back, buttocks, arms, and legs, with the largest spot measuring 10x10 cm. Additionally, the child exhibited coarse facial features, a high-arched palate, low-set ears, and a depressed nasal bridge (Figure 1). 

During the systemic examination, hepatosplenomegaly was noted (liver was 3 cm below the right costal margin and spleen was 4.5 cm below the left costal margin). Cranial nerve examination, reflexes, and tone were normal. Heart sounds S1 and S2 were heard with no murmurs. Clear breath sounds were heard bilaterally upon auscultation. The child's height (67 cm) and weight (7.180 kg) were at the 1st percentile, while his head circumference (47.5 cm) was at the 90th percentile. Additionally, his mid-arm circumference was measured at 14.5 cm. On fundus examination, the cornea appeared slightly hazy, while the lens and optic disc appeared normal. No cherry red spot was present.

Histopathological examination of the skin lesions revealed pigmented melanocytes in the dermis, confirming the diagnosis of Mongolian spots. The urine glycosaminoglycan test was positive, confirming mucopolysaccharidosis. Magnetic resonance imaging of the brain was normal.

The presence of multiple aberrant Mongolian spots, coarse facial features, a hazy cornea, and a positive urine glycosaminoglycan test prompted us to conduct further research, prioritizing lysosomal storage disorders.

**Figure 2:**
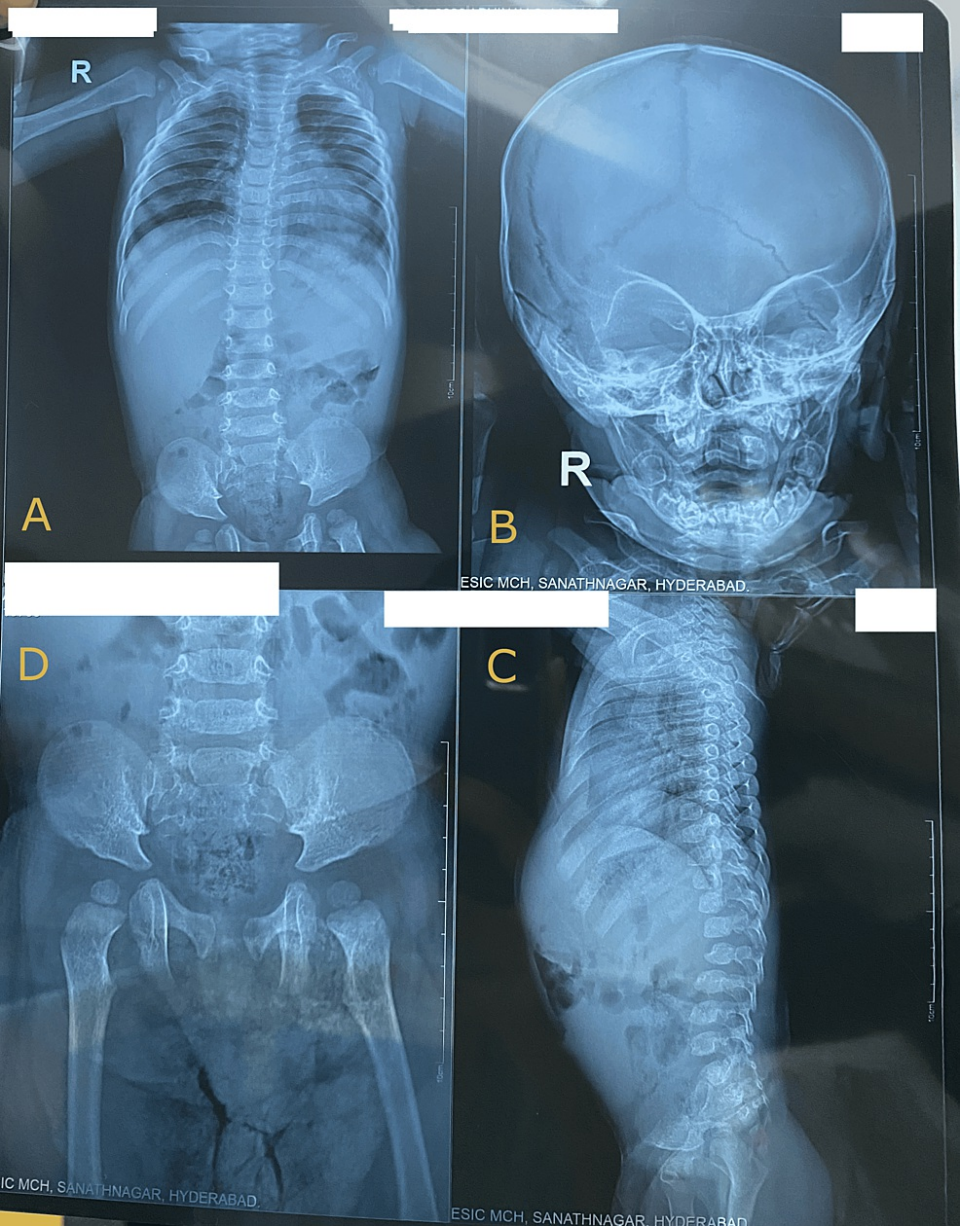
Oar-shaped anterior ribs, cardiomegaly, and hepatosplenomegaly on chest X-ray (A), macrocephaly on skull X-ray (B), horizontal acetabular roof on hip X-ray (D), anteroinferior beaking of the lumbosacral vertebral bodies and increased interpedicular distance in lumbosacral region on spine X-ray (C).

**Figure 3:**
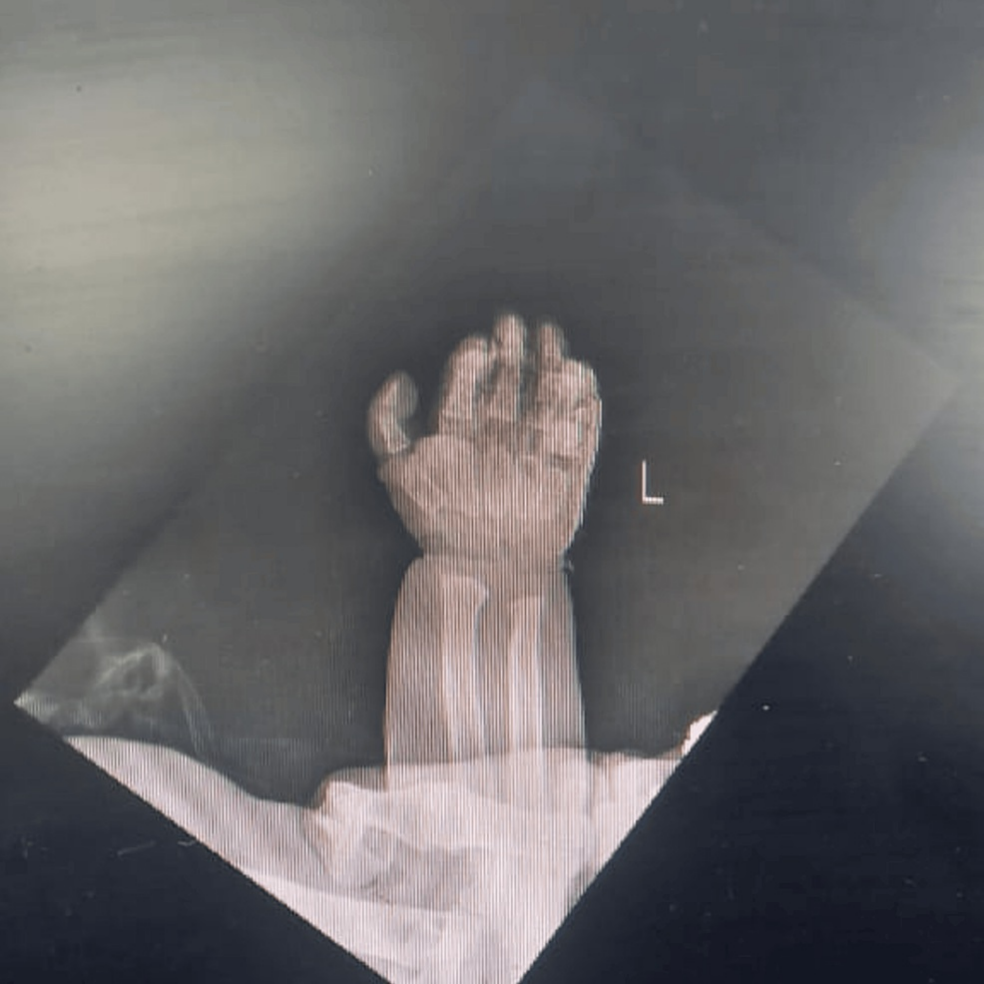
Bullet-shaped proximal phalanges and metacarpals in the hand.

X-rays revealed oar-shaped anterior ribs, cardiomegaly, and hepatosplenomegaly in the chest; macrocephaly and patent sutures in the skull; a horizontal acetabular roof in the hip; anteroinferior beaking of lumbosacral vertebral bodies and increased interpedicular distance in the lumbosacral region in the spine (Figure 2); and bullet-shaped proximal phalanges and metacarpals in the hand (Figure 3). Abdominal ultrasound confirmed hepatosplenomegaly. 2D echocardiography revealed left ventricular hypertrophy.

The MPS spot test was positive, and electrophoresis for MPS revealed bands for chondroitin sulfate and dermatan sulfate, confirming the diagnosis of MPS. Enzyme assay revealed no alpha-iduronidase activity and normal beta-galactosidase activity, thus confirming Hurler's disease.

## Discussion

Atypical Mongolian spots may be a cutaneous sign of underlying inborn errors of metabolism and neurocristopathies [4]. Human keratinocytes and dermal fibroblasts produce nerve growth factor (NGF), a crucial signal for transdermal melanocyte migration. NGF exerts its effects through the Trk protein (tyrosine kinase type receptor) and receptors found on melanocytes. In cases of inborn errors of metabolism, accumulated metabolites bind to the Trk protein through glycosylation, resulting in abnormal NGF activity. This aberrant activity can disrupt melanocyte migration, as melanocytes also possess NGF receptors. Additionally, metabolite-Trk binding triggers melanin synthesising pathways in dormant melanocytes [3].

Kikuchi noted that while there were racial disparities in Mongolian spot expression, all newborns, regardless of race, had dermal melanocytes in their buttocks upon microscopic examination. He observed that the main distinction lay in the melanocyte quantity, which was lower in normally appearing buttocks compared to pigmented ones. Additionally, melanocytes in white children contained inactive, incompletely melanised melanosomes. The difference could also stem from the duration of dermal melanocyte production, which was longer in Asians compared to Caucasians. Consequently, birthmarks would persist at birth in the former but would have faded in the latter, contributing to a higher prevalence at birth among Asians [8].

Mongolian spots are classified into three types based on regression: Common type - these spots typically regress by early childhood; Extensive type - these spots regress at a very slow pace; Persistent type - these spots may persist into adulthood [9].

Mongolian spots are further classified as follows: Atypical Mongolian Spots - Lesions appearing in areas beyond the typical locations, such as the upper back, face, legs, and chest. These lesions may persist and occur in multiple areas. Recent studies have noted the occurrence of atypical Mongolian spots along the distribution of the ophthalmic, mandibular, and maxillary areas on the face [10,11,12]. Persistent Mongolian spots - Lesions persisting beyond childhood into adulthood. Extra-sacral, dark, large (>10 cm), multiple, and widespread Mongolian spots tend to persist longer. Widespread Mongolian spots - Lesions distributed extensively across the body. Superimposed Mongolian spots - A darker pigmented Mongolian spot appearing over another Mongolian spot. This may result from two separate waves of melanocyte migration or melanocyte arrest at two dermal levels during migration. Speckled Mongolian spots - Mottled or dotted grey-blue macules of varying shades. Acquired Mongolian spots - Lesions appearing later in life, often attributed to the activation of dormant dermal melanocytes. Progressive Mongolian spots - Lesions increasing in number, with new areas affected beyond the early months of life [2].

In recent years, the documentation of Mongolian spots has gained significance in medico-legal contexts due to the potential confusion with bruises, particularly when they occur in atypical locations [6]. This confusion can result in misdiagnosis of child abuse or battered baby syndrome. Mongolian spots can be differentiated from bruises as they are non-tender, do not change colour or evolve over time, and may take several months to fade away [3].

Considering the typically self-limiting nature of this condition, active intervention is generally unnecessary. While Q-switch lasers (ruby, alexandrite, and neodymium-doped yttrium aluminium garnet) and intense pulsed light have been employed in cases of persistent lesions, outcomes have been uncertain, particularly in adults and sacral Mongolian spots. Therefore, laser treatment for persistent spots should ideally commence before the age of 20 [2]. MPS exhibit positive responses to stem cell transplantation or enzyme replacement therapy when initiated at an early stage [3]. Recent studies have shown that bone marrow transplantation in early childhood can lead to positive outcomes in Hurler's disease, including the reversal of clinical features [13,14].

## Conclusions

Mongolian spots, although considered benign in nature, should raise suspicion for underlying inborn errors of metabolism if they persist beyond infancy or appear atypically. They aid physicians in the early detection of associated disorders, enabling early interventions such as genetic counselling, enzyme replacement therapy, and symptomatic treatment for associated clinical features. Bone marrow transplantation is the ultimate treatment of choice, replacing stem cells, and could be highly beneficial if the diagnosis is made early. Further research is needed in this area.
